# Supplementary material for: Correction: Morphology and histology of vom Rath's organ in brush-footed butterflies (Lepidoptera: Nymphalidae)
Source: PLoS One. 2020 Sep 24;15(9):e0239885. doi: 10.1371/journal.pone.0239885 (PMC7514025; doi:10.1371/journal.pone.0239885)
Supplement: S2 File — (PDF) [file pone.0239885.s002.pdf]

PLOS ONE

RESEARCH ARTICLE

Morphology and histology of vom Rath's organ in brush-footed butterflies (Lepidoptera: Nymphalidae)

Joel Lastra-Valdés[1,2], José Roberto Machado Cunha da Silva[3], Marcelo Duarte[2,4]*

1 Museo Nacional de Historia Natural de Cuba, Havana, Cuba, **2** Programa de Pós-Graduação em Sistemática, Taxonomia Animal e Biodiversidade, Museu de Zoologia, Universidade de São Paulo, São Paulo, São Paulo, Brazil, **3** Departamento de Biologia Celular e do Desenvolvimento, Laboratório de Histofisiologia Evolutiva, Instituto de Ciências Biomédicas, Universidade de São Paulo, São Paulo, São Paulo, Brazil, **4** Department of Entomology, Research Associate, National Museum of Natural History, Smithsonian Institution, Washington, District of Columbia, United States of America

* mduartes@usp.br

Check for updates

OPEN ACCESS

Citation: Lastra-Valdés J, Silva JRMCd, Duarte M (2020) Morphology and histology of vom Rath's organ in brush-footed butterflies (Lepidoptera: Nymphalidae). PLoS ONE 15(4): e0231486. https://doi.org/10.1371/journal.pone.0231486

Editor: Feng ZHANG, Nanjing Agricultural University, CHINA

Received: December 17, 2019

Accepted: March 24, 2020

Published: April 23, 2020

Copyright: © 2020 Lastra-Valdés et al. This is an open access article distributed under the terms of the Creative Commons Attribution License, which permits unrestricted use, distribution, and reproduction in any medium, provided the original author and source are credited.

Data Availability Statement: All relevant data are within the manuscript.

Funding: JLV, Bolsa de Mestrado Demanda Social, Coordenação de Aperfeiçoamento de Pessoal de Nível Superior (CAPES), http://capes.gov.br/ MD, 2002/13898-0 and 2016/50384-8, Fundação de Amparo à Pesquisa do Estado de São Paulo, http://fapesp.br/ MD, 440597/2015-3, PROTAX II, Coordenação de Aperfeiçoamento de Pessoal de Nível Superior (CAPES), http://capes.gov.br/ MD, 312190/2018-2 and 426914/2018-0, Conselho Nacional de Desenvolvimento Científico e

Abstract

Vom Rath's organ, located at the distal end of the third segment of the labial palp, is one of the recognized synapomorphies of Lepidoptera (Insecta). Information about the structural and histological morphology of this organ is sparse. The structure of vom Rath's organ in four species of Nymphalidae, three frugivorous: *Fountainea ryphea* (Charaxinae: Anaeini), *Morpho helenor achillaena* (Satyrinae: Morphini) and *Hamadryas epinome* (Biblidinae: Ageroniini), and the nectarivorous species *Aeria olena* (Danainae: Ithomiini) is described by means of scanning electron microscopy and histology. The species showed significant differences in the cavity shape, setal morphology and arrangement, opening shape and location, associated with the organization of cell groups, type of axon, and degree of development. These differences do not seem to be related to feeding habit. No cell groups were found in *Actinote thalia* (Heliconiinae: Acraeini) and *Heliconius erato phyllis* (Heliconiinae: Heliconiini), and for the first time the absence of vom Rath's organ is documented in the clade Ditrysia. A terminology is proposed to improve understanding of the organ morphology, with an extensive analysis of the previous descriptions.

Introduction

The monophyly of Lepidoptera is well supported by 24 synapomorphies [1]. Among these is a depression on the distal segment of the labial palps, known as vom Rath's organ. The pioneer works of Hicks [2] and vom Rath [3, 4] reported that the form of the organ varies among species and genera. Both authors mentioned that they analyzed a large number of species, but described few examples: *Acherontia atropos* (Linnaeus, 1758) (Sphingidae), *Malacosoma neustria* (Linnaeus, 1758) (Lasiocampidae), *Argynnis paphia* (Linnaeus, 1758) (Nymphalidae: Heliconiinae: Argynnini), and an unidentified geometrid [2]; and *A. atropos*, *Agrius convolvuli* Linnaeus, 1758 (Sphingidae), and *Pieris* sp. (Pieridae: Pierinae) [4]. The organ is mentioned in a few other general studies of adult morphology, although without detailed descriptions; see Orfila [5] for *Archaeoprepona demophoon* (Hübner, [1814]) and *A. chromus* (Guérin-

Tecnológico, http://cnpq.br All authors thank CAPES, CNPq and FAPESP for the financial support. The funders had no role in study design, data collection and analysis, decision to publish, or preparation of the manuscript.

Competing interests: The authors have declared that no competing interests exist.

Méneville, [1844]) (Nymphalidae); Duarte et al. [6] for *Hemiargus hanno* (Stoll, 1790) (Lycaenidae); Bizarro et al. [7] for [*Thyridia psidii cetoides* (Rosenberg & Talbot, 1914) (Nymphalidae); Mielke et al. [8] for *Zaretyis ithys itylus* (Westwood, 1850) and *Prepona claudina anneta* (G. Gray, 1832) (Nymphalidae); Camargo et al. [9] for *Almeidaia aidae* Mielke & Casagrande, 1981 (Saturniidae); and Leite et al. [10] for *Heraclides anchisiades capys* (Hübner, [1809]) (Papilionidae).

Descriptions of the organ in non-ditrysian lepidopterans are brief and usually without illustrations. Examples include descriptions of members of Mnesarchaeidae [11], Neopseustidae [11, 12, 13, 14], Micropterigidae [15, 16], Incurvariidae [17], Palaephatidae [18], Heterobathmiidae [19], Agathiphagidae [16], and Andesianidae [20]. However, the depth, degree of development, and even the presence of the cavity seem to be quite diverse among families, once these descriptions are analyzed. No comparative studies have been done with these lineages.

For ditrysian species, descriptions of vom Rath's organ are more detailed, generally based on images obtained with the aid of scanning and transmission electron microscopy. These include five tineid species [21], *Pieris rapae* (Linnaeus, 1758) (Pieridae) [22], *Manduca sexta* (Linnaeus, 1763) (Sphingidae) [23], three species of *Amerila* (*Rhodogastria*) (Erebidae) [24], *Homoeosoma nebulella* (Denis & Schiffermüller, 1775) (Pyralidae) [25], *Cactoblastis cactorum* (Berg, 1885) (Pyralidae) [26], *Helicoverpa armigera* (Hübner, 1805) (Noctuidae) [27], *Mythimna separata* (Walker, 1865) (Noctuidae) [28], *Carposina sasakii* Matsumura, 1900 (Carposinidae) [29], and *Grapholita molesta* (Busck, 1916) (Tortricidae) [30].

An analysis of descriptions of the organ's cavity shows that it has different forms, although authors usually refer to it as bottle-shaped [3, 4, 23, 29, 31]. The number and morphology of the sensilla are also variable. Occasionally, two regions are reported in the cavity, depending on the morphology of the setae [29, 30, 32]: an internal region covered with sensilla, and another region near the opening, with microtrichia or piliform setae [4, 24].

In view of the importance of synapomorphies as a basis for phylogeny and evolution [31], it is believed that vom Rath's organ can provide phylogenetically informative data [1, 32]. However, the lack of anatomical and histological descriptions hinders its use in these studies. Here, we describe the structure and histology of vom Rath's organ in six species of Neotropical Nymphalidae (Lepidoptera: Ditrysia: Papilionoidea), representing the frugivorous and nectarivorous habits observed in the family. The occurrence of vom Rath's organ was also investigated in other species phylogenetically related to those studied in the present work.

Materials and methods

Material studied

Six species were chosen to describe the structural and histological morphology of vom Rath's organ: frugivorous: *Fountainea ryphea* (Cramer, 1775) (Charaxinae: Anaeini) (specimens MZSP 43343–43347, 43358–43362), *Morpho helenor achillaena* (Hübner, [1819]) (Satyrinae: Morphini) (specimens MZSP 13966, 16758–16759, 44229–44236), *Hamadryas epinome* (Felder & Felder, 1867) (Biblidinae: Ageroniini) (specimens MZSP 43322–43331, 43334, 43336); and nectarivorous: *Aeria olena* Weymer, 1875 (Danainae: Ithomiini) (specimens MZSP 10310, 14660–14662, 15693, 16802, 16804, 44238–44240), *Actinote thalia* (Linnaeus, 1758) (Heliconiinae: Acraeini) (specimens MZSP 44225, 44226) and *Heliconius erato phyllis* (Fabricius, 1775) (Heliconiinae: Heliconiini) (specimens MZSP 44227, 44228). All the species are common in southeastern Brazil [33] and can be easily collected in the warmer months of the year. All specimens studied are deposited in the Lepidoptera Collection of the Museu de Zoologia da Universidade de São Paulo (it is a public institution and specimens deposited here are permanently accessible by others).

The right palp of each of ten individuals per species, five of each sex (except *A. thalia* and *H. erato phyllis*, see results section), from the Lepidoptera Collection of the Museu de Zoologia da Universidade de São Paulo (São Paulo, SP, Brazil) (MZUSP) was removed and photographed under a stereomicroscope with an attached Zeiss AxioCam MRc5 camera. Photographs (12–15 per piece) were obtained with the support of AxioVision software rel. 4.8 and then assembled with the use of CombineZP software. Palp total length was estimated from the photographs using TPS software [34].

The occurrence of the organ was also investigated in six additional heliconiine species, based on dried material deposited in the MZUSP Lepidoptera Collection: *Actinote parapheles* Jordan, 1913 (specimens MZSP 44219, 44220), *Agraulis vanillae* (Linnaeus, 1758) (specimens MZSP 44223, 44224), *Argynnis paphia* (Linnaeus, 1758) (specimens MZSP 44221, 44222), *Euptoieta hegesia* (Cramer, 1779) (specimens MZSP 18408, 20767), *Heliconius sara* (Fabricius, 1793) (specimens MZSP 15048, 42249), and *Philaethria wernickei* (Röber, 1906) (specimens MZSP 14459, 16229).

Material collection and fixation

Four field expeditions were carried out at the Reserva Biológica Serra do Japi, Municipality of Jundiaí, São Paulo State, Brazil–protected area (23˚14' S, 46˚56' W; 1,049 m a.s.l.) (permit provided by Fundação Serra do Japi—process number 004/2018, and Instituto Chico Mendes de Conservação da Biodiversidade/ Ministério do Meio Ambiente—process number 10430–10), and two others at the Santa Genebra Mata transmission line, Parque Estadual Intervales, municipalities of Guapiara (24˚12' S, 48˚30' W; 809 m a.s.l.) and Apiaí (21˚24' S, 48˚45' W; 905 m a.s.l.), São Paulo State, Brazil–protected areas (permit provided by Instituto Chico Mendes de Conservação da Biodiversidade/ Ministério do Meio Ambiente–process number 820/2017). Frugivorous species were collected with attraction traps (Van Someren-Rydon model) using baits composed of mixtures of decomposing banana and sugarcane juice. Nectarivorous species were actively sampled with entomological nets. We did not collect endangered or protected species.

Individuals were killed by thoracic compression and their labial palps were removed, except for specimens of *A. olena* in which, due to their small size, the entire heads were removed. Dead individuals with the palps removed, or in some case decapitated were deposited in the MZUSP Lepidoptera Collection. The labial palps and heads were placed in individual vials and fixed in modified McDowell's solution [35] (2.0 glutaraldehyde, 4.0 paraformaldehyde in 0.1 M PBS, pH = 7.2) for 24 h. Then, the pieces were passed through a diluted ethanol series (10%, 30%, 50% and 70%), remaining 1 h in each dilution. Tissues were stored in individual vials with 70% ethanol until laboratory processing.

Histology of vom Rath's organ

The histological procedures were carried out in the Laboratório de Histofisiologia Evolutiva of the Departamento de Biologia Celular e do Desenvolvimento, Instituto de Ciências Biomédicas, Universidade de São Paulo. Pieces in 70% ethanol were passed through an ascending ethanol dilution series, remaining 30 min in each dilution (70%, twice in 90%, and twice in 100%). Next, they were immersed in a historesin and ethanol mixture (1: 1) for 4 h, left in pure resin overnight, and finally placed in resin with hardener for 48 h at 37˚C. The resulting blocks were mounted to allow sectioning in the sagittal plane and were sectioned with a microtome (American Optical Company, model 820), using glass knives. The sections (3 μm thickness) were stretched in cold water mixed with ethanol, mounted on microscope slides, and stained with a solution of 1% toluidine blue and 0.5% acid fuchsin. A light microscope (Carl Zeiss Axio Scope A1) was used to scan the images with the support of the software Zen2012 from Carl Zeiss (Blue Edition).

Description of the organ form and SEM

After the scales were removed and before a palp was prepared for scanning electron microscopy (SEM), it was immersed in alcohol hand sanitizer gel, placed on a well slide, and observed under a Leica DM 750 light microscope to observe the form of the organ in the cleared palp. Photographs were taken with the support of Leica Application Suite software, version 4.4.0. The photographs were used to measure the depth of the organ cavity and the length of the distal palpomerus, with the use of TPS software [34]. In order to normalize the comparisons among species, we established two indexes of organ development or relative size: the ratio of the cavity depth to the palpomerus, and of the cavity depth to the entire length of the palp.

To analyze the structure of the labial palps, we removed their scales with the use of minuten pins and adhesive tape. The labial palps were studied using two protocols: i) the distal palpomerus (with the scales removed) was assembled intact on the SEM specimen stub, to observe the opening of the organ (size and shape) in all species; and ii) the distal palpomerus was sectioned transversely in the middle of the organ with a common razor blade attached to a mechanical pencil, to observe and describe the inner setae and microtrichia. This latter procedure was done only for *F. ryphea* and *M. helenor achillaena*, which have relatively large organs that were easier to manipulate with the available instruments.

Both types of preparations were run through an ascending ethanol series, remaining 30 min in each dilution (70%, 90%, and 100%), and then immersed for 30 min in hexamethyldisilazane for final dehydration, thereby eliminating the need for critical-point drying [36]. The preparations were placed on SEM specimen stubs with double-sided tape and metallized in gold prior to observation with a Carl Zeiss LEO 440 scanning electron microscope in the Laboratório de Microscopia Eletrônica de Varredura at the Museu de Zoologia da USP, and a Zeiss DSM 940 at the Laboratório de Microscopia Eletrônica de Varredura in the Instituto de Biociências da USP.

Results

The morphometric measurements of the palp, distal palpomerus, and depth of the cavity in all species are provided in Table 1.

The study of optical microscopy is illustrated in Figs 1–12, that of SEM in Figs 13–28, and of histology in Figs 29–36.

Frugivorous species

***Fountainea ryphea* (Charaxinae: Anaeini) (Figs 1, 13, 14, 29 and 30).** The vom Rath's organ is conspicuous once the scales are removed (Fig 1). Its opening is ovoid (Fig 13), subterminal and slightly ventral, and covered with a dorsal lobe. In males the largest opening diameter was

Table 1. Morphometric means of vom Rath's organ and the indexes of development for four nymphalids.

Species and sex	Palp Length (μm)	Distal Palpomerus Length (μm)	Cavity depth (μm)	Cavity:Palp (%)	Cavity:Distal palpomerus (%)
Fountainea ryphea ♂	5760.0 ± 496.0 (n = 5)	465.0 ± 53.9 (n = 4)	158.1 ± 22.5 (n = 4)	2.74	33.99
Fountainea ryphea ♀	5874.0 ± 451.2 (n = 5)	551.0 ± 58.6 (n = 4)	198.3 ± 4.5 (n = 4)	3.38	35.97
Morpho helenor ♂	7054.0 ± 400.8 (n = 5)	1075.4 ± 94.9 (n = 6)	180.6 ± 34.0 (n = 4)	2.56	17.10
Morpho helenor ♀	8108.0 ± 594.4 (n = 5)	1056.3 ± 87.4 (n = 5)	No data	No data	No data
Hamadryas epinome ♂	5525.0 ± 205.0 (n = 5)	1095.5 ± 65.6 (n = 7)	120.2 ± 1.2 (N = 2)	2.18	10.97
Hamadryas epinome ♀	5430.0 ± 380.0 (n = 5)	1072.7 ± 50.9 (n = 5)	101.2 ± 8.9 (n = 3)	1.90	9.43
Aeria olena ♂	2506.0 ± 187.2 (n = 5)	114.9 ± 4.3 (n = 3)	38.5 ± 0.02 (n = 3)	1.53	33.50
Aeria olena ♀	2170.0 ± 160.0 (n = 5)	107.0 (n = 1)	No data	No data	No data

https://doi.org/10.1371/journal.pone.0231486.t001

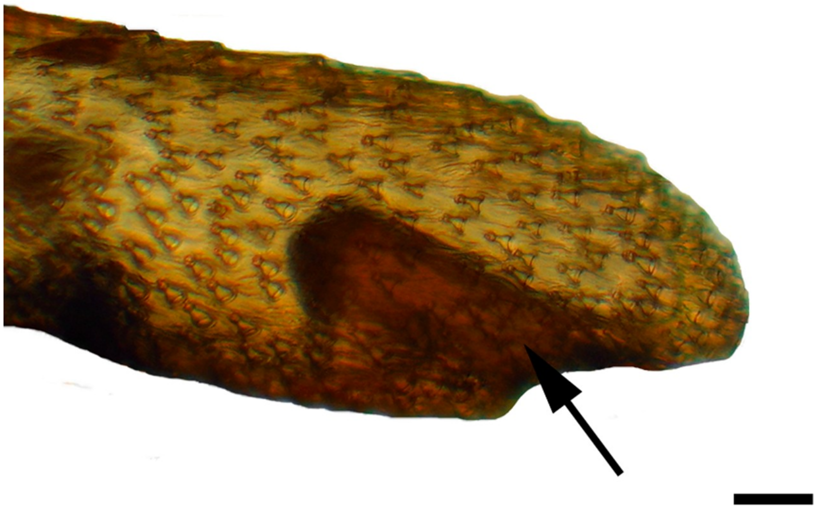

Fig 1. Distal palpomerus. *Fountainea ryphea* (Cramer, 1775) (Charaxinae: Anaeini) (♀), black arrow indicates vom Rath's organ, scale bar 50 μm.

https://doi.org/10.1371/journal.pone.0231486.g001

37.78 ± 6.20 μm (n = 4); in the two females measured the opening diameters were 44.90 and 44.80 μm, respectively. The smallest diameter in males was 27.43 ± 3.59 μm (n = 4), and the smallest diameters in the two females were 37.37 and 35.50 μm. The form was bottle-shaped, i.e., with a neck or tube near the opening, which increases slightly in diameter toward the inside up to approximately half the length of the organ, where the cavity abruptly widens to form an atrium approximately twice as wide as the opening and the neck. Piliform and flat microtrichia (approximately 150) directed toward the opening densely cover the inner surface but do not extend outside the tube. Slightly club-shaped, smooth coeloconic setae (approximately 100) occupy the atrium (Fig 14). Cell groups around the atrium are connected to an axon that runs ventrally and fuses with the cuticle (Figs 29 and 30).

***Morpho helenor achillaena* (Satyrinae: Morphini) (Figs 2, 15, 16, 31 and 32).** The vom Rath's organ is conspicuous once the scales are removed (Fig 2). The opening is circular (Fig 15) and terminal. The mean diameter of the opening in males was 42.20 ± 4.67 μm (n = 3); no

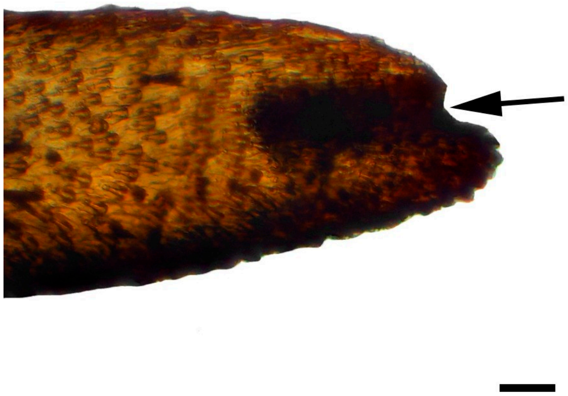

Fig 2. Distal palpomerus. *Morpho helenor achillaena* (Hübner, [1819]) (Satyrinae: Morphini) (♂), black arrow indicates vom Rath's organ, scale bar 50 μm.

https://doi.org/10.1371/journal.pone.0231486.g002

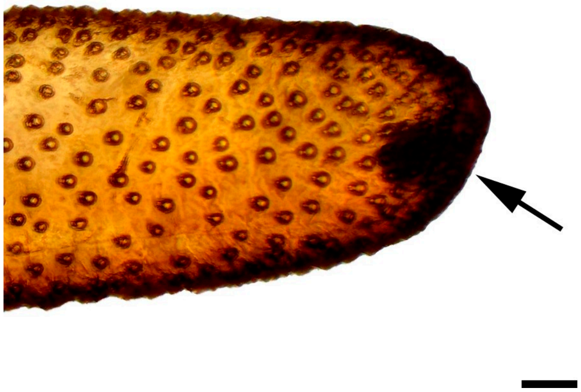

Fig 3. Distal palpomerus. *Hamadryas epinome* (Felder & Felder, 1867) (Biblidinae: Ageroniini) (♂), black arrow indicates vom Rath's organ, scale bar 50 μm.

https://doi.org/10.1371/journal.pone.0231486.g003

females were measured. The organ is drop-shaped, i.e., the largest diameter of the cavity is slightly larger than the aperture and is located approximately midway between the opening and the bottom of the cavity. Piliform and flat microtrichia (between 150 and 200) densely cover the region between the opening and the largest diameter of the cavity. All these microtrichia point toward the opening and extend outside it, and some of them are bifurcated. The bottom of the cavity is covered with 30 to 40 slightly club-shaped, smooth coeloconic setae (Fig 16). The cell groups are located around the atrium and connected to an axon that abruptly fuses with the cuticle (Figs 31 and 32).

Hamadryas epinome **(Biblidinae: Ageroniini) (Figs 3, 17, 18 and 33).** The vom Rath's organ is relatively small (Fig 3). Its opening is circular (Fig 17), located in terminal position, with the diameter 27.51 μm in the male (n = 1) and 25.22 μm in the female measured (n = 1). The organ is sacculiform (Fig 18). It was not possible to section the structure to obtain SEM images, due to the small size of the distal palpomerus. Piliform microtrichia are present at the opening of the organ, but do not extend outside it (Fig 17). The cell groups surround most of the inner wall. No axon was observed (Fig 33).

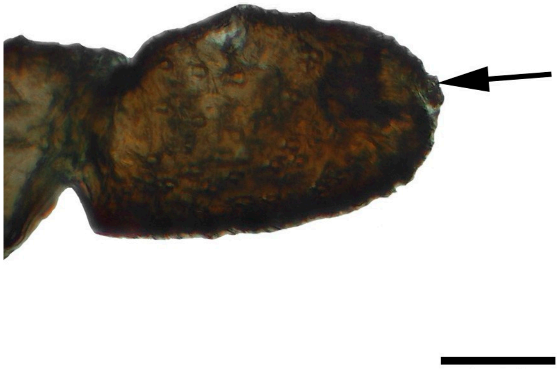

Fig 4. Distal palpomerus. *Aeria olena* Weymer, 1875 (Danainae: Ithomiini) (♂), black arrow indicates vom Rath's organ, scale bar 50 μm.

https://doi.org/10.1371/journal.pone.0231486.g004

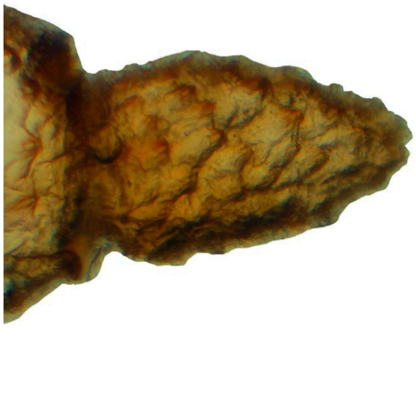

Fig 5. Distal palpomerus. *Actinote thalia* (Linnaeus, 1758) (Heliconiinae: Acraeini) (♀), scale bar 50 μm.

https://doi.org/10.1371/journal.pone.0231486.g005

Nectarivorous species

***Aeria olena* (Danainae: Ithomiini) (Figs 4, 19 and 34).** Due to the small size of the distal palpomerus, it was not possible to section the vom Rath's organ, but analysis under the optical microscope indicated that it is well developed and probably sacculiform (Fig 4). The opening is circular, obstructed by flat microtrichia that do not extend outside it (Fig 19). The opening diameter was 23.25 μm for the male (n = 1) and 22.59 μm for the female (n = 1). A well-developed cell group is connected to an axon that is apparently not attached to the cuticle (Fig 34).

***Actinote thalia* (Figs 5, 20 and 35) and *A. parapheles* (Figs 6 and 21) (Heliconiinae: Acraeini).** The vom Rath's organ is absent in both sexes of *A. thalia* (Fig 5). There is no depression of the cuticle in the region where the opening of the organ is normally located (Fig 20). No cell groups were observed in the histological sections (Fig 35). The absence of the organ was also confirmed for both sexes of *A. parapheles* (Figs 6 and 21).

***Heliconius erato phyllis* (Heliconiinae: Heliconiini) (Figs 7, 22 and 36), other Heliconiini (Figs 8–10, 23 and 24) and Argynnini (Figs 11, 12 and 25–28).** The vom Rath's organ is absent in both sexes (Fig 7). There is no depression of the cuticle in the region where the opening of the organ is normally located (Fig 22). No cell groups were observed in the histological sections (Fig 36). As in *Actinote*, the absence of the organ was confirmed in other species of Heliconiini:

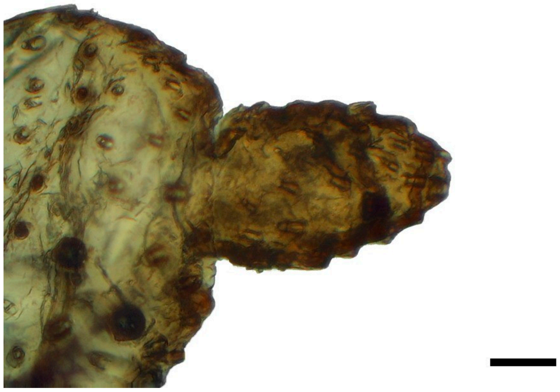

Fig 6. Distal palpomerus. *Actinote parapheles* Jordan, 1913 (Heliconiinae: Acraeini) (♂), scale bar 50 μm.

https://doi.org/10.1371/journal.pone.0231486.g006

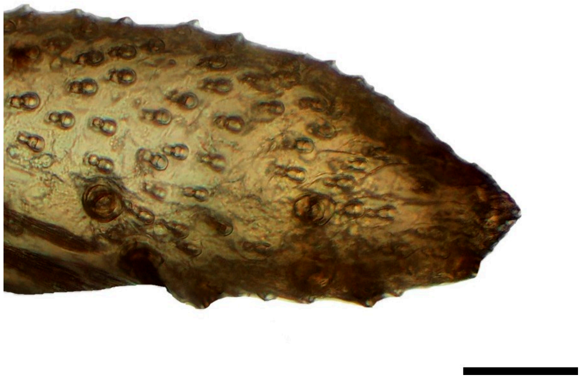

Fig 7. Distal palpomerus. *Heliconius erato phyllis* (Fabricius, 1775) (Heliconiinae: Heliconiini) (♀), scale bar 50 μm.

https://doi.org/10.1371/journal.pone.0231486.g007

H. *sara*, *A*. *vanillae* and *P*. *wernickei* (**Figs 8–10, 23 and 24**). However, the organ is present in two species of the tribe Argynnini, *A*. *paphia* and *E*. *hegesia* (**Figs 11, 12 and 25–28**).

Discussion and conclusions

The degree to which vom Rath's organ may be phylogenetically informative remains unknown. However, the present finding that the organ is absent in phylogenetically related groups (Acraeini and Heliconiini) is quite stimulating. Additional taxa must be included in future analyses. As a synapomorphy, this organ constitutes a novel evolutionary step for Lepidoptera, but there is no well-founded hypothesis about the possible selective pressures involved. How variable is its morphology among lepidopteran clades? Why is it missing in some groups? It is recognized that vom Rath's organ is involved in CO_2 detection, but why and how is it important for survival? Further studies are needed, such as that of Stange et al. [26] for selection of oviposition sites, and those of Guerenstein et al. [37] and Thom et al. [38] for selection of nectar sources. It is also necessary to determine if vom Rath's organ is involved in detecting other kinds of stimulus. The present study increased our knowledge of the morphology of vom Rath's organ in some nymphalid butterflies, proposing a terminology for future morphological work, and discussing the previous studies of this structure.

Morphological patterns

In view of the few descriptions of vom Rath's organ, a synapomorphy for Lepidoptera, any advance is useful for evaluating how informative this structure might be in systematic studies.

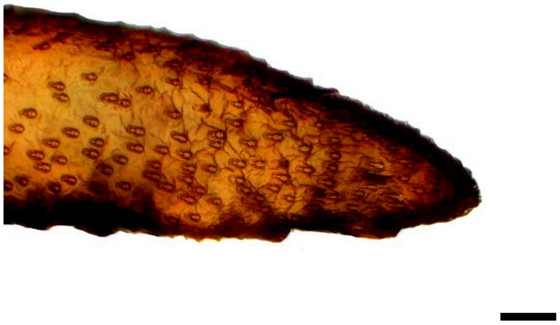

Fig 8. Distal palpomerus. *Heliconius sara* (Fabricius, 1793) (Heliconiinae: Heliconiini) (♂), scale bar 50 μm. Scale bars 50 μm. Black arrows indicate vom Rath's organ.

https://doi.org/10.1371/journal.pone.0231486.g008

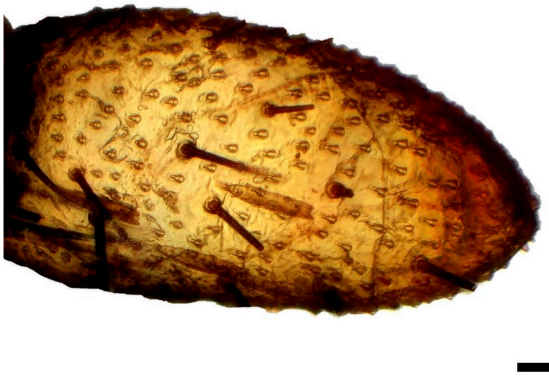

Fig 9. Distal palpomerus. *Agraulis vanillae* (Linnaeus, 1758) (Heliconiinae: Heliconiini) (♀), scale bar 50 μm.

https://doi.org/10.1371/journal.pone.0231486.g009

Counting our results, there is more-or-less detailed information on the morphology of vom Rath's organ for 23 dytrisian species belonging to 11 families (Carposinidae, Erebidae, Geometridae, Lasiocampidae, Noctuidae, Nymphalidae, Pieridae, Pyralidae, Sphingidae, Tineidae and Tortricidae).

There is also a lack of a standardized terminology. Vom Rath's organ is a simple cuticular invagination at the tip of the distal palpomerus, forming a cavity with sensilla connected to sensory cells at the deepest part of the cavity wall. The form of the invagination was not described by Stange et al. [26], Song et al. [30], Bogner [39], or Stange [40]; or was erroneously reported as bottle-shaped by Kent et al. [23], Chen & Hua [29], and Zhao et al. [27]. For this purpose, we consider as a bottle-shaped form only the cases of the unidentified geometrid reported by Hicks [2]; "*Pieris* sp.", probably *P. rapae* or *P. brassicae* [4]; and *F. ryphea* (Fig 14). In these cases, the invagination forms a wide cavity or atrium at its deepest portion. This wide and deep atrium communicates with the exterior through a narrower neck with a diameter equal or subequal to the opening.

We also propose the terms sacculiform, fusiform, drop-shaped, tubular and tapered to describe these forms. Sacculiform vom Rath´s organs have a wide atrium similar to the bottle-shaped ones, but they either do not have a neck or the neck is very short. This seems to be the case for *Acherontia atropos* and *Agrius convolvuli* (Sphingidae), according to the descriptions of vom Rath [4], and is clearly the case for *Amerila* (*Rhodogastria*) sp. (Erebidae) [24], and *Hamadryas epinome* (Fig 18). Another example, but with some differences, is *Manduca sexta* (Sphingidae), in which the cavity has two different atria separated by a furrow [23]. Both the fusiform and drop-shaped forms have an atrium without a neck, and with the maximum width about half of the length. If the bottom of the atrium is pointed, then the format is

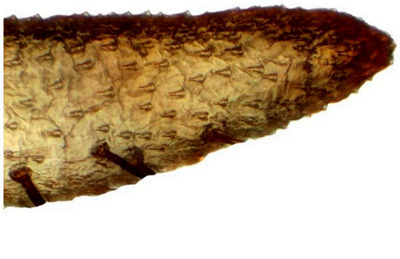

Fig 10. Distal palpomerus. *Philaethria wernickei* (Röber, 1906) (Heliconiinae: Heliconiini) (♂), scale bar 50 μm.

https://doi.org/10.1371/journal.pone.0231486.g010

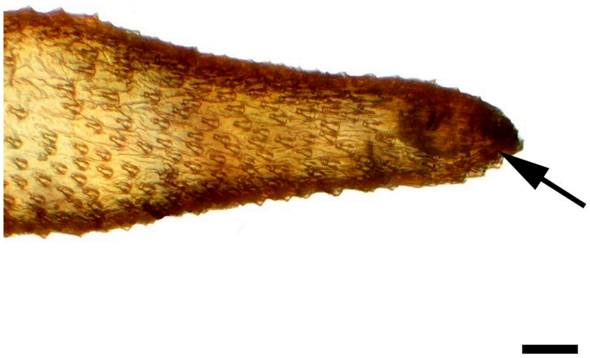

Fig 11. Distal palpomerus. *Argynnis paphia* (Linnaeus, 1758) (Heliconiinae: Argynnini) (♀), black arrow indicates vom Rath's organ, scale bar 50 μm.

https://doi.org/10.1371/journal.pone.0231486.g011

fusiform, as in *Carposina sasakii* (Carposinidae) [29]. If the bottom of the atrium is rounded, then the form is drop-shaped, as in *Morpho helenor achillaena* (Fig 16). Tubular vom Rath's organs have more or less the same width from the opening to the bottom of the cavity, so they do not form an atrium; examples are *Argynnis paphia* (Nymphalidae: Heliconiinae) [2] and *Cactoblastis cactorum* (Pyralidae) [26]. Finally, tapered vom Rath's organs have an opening wider than the bottom of the cavity, forming a funnel, but not an atrium, as in *Helicoverpa armigera* [27] and *Mythimna separata* (Noctuidae) [28].

Another recognizable pattern in various ditrysians is the morphological and positional differentiation of the setae. These are present in two forms (erroneously defined as three by Song et al. [30]); generally the inner setae are more or less clubbed, with grooved walls, while those closer to the opening are piliform or laminar with smooth walls [24, 26, 27, 29, 30]. *Fountainea ryphea* and *M. helenor achillaena* have this pattern of differentiation in the morphology and arrangement of both types of setae (see Figs 14 and 16). Their inner setae are coeloconic sensilla and are connected to the sensory cells. Probably the same is true for *H. epinome* (Figs 17 and 33). Histological imaging shows that the cell groups are always related to the inner regions of the cavity (Figs 29,30,31,32,33,34). In contrast, the more-distal setae are not related to cell groups at all, and we classified them as microtrichia. Originally, vom Rath [4] noted this difference and considered that these "oblique hairs toward the opening" protect the structure from the external environment. Therefore, we can divide the organ cavity into two morpho-

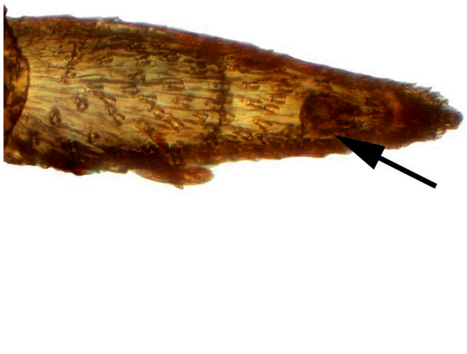

Fig 12. Distal palpomerus. *Euptoieta hegesia* (Cramer, 1779) (Heliconiinae: Argynnini) (♂), black arrow indicates vom Rath's organ, scale bar 50 μm.

https://doi.org/10.1371/journal.pone.0231486.g012

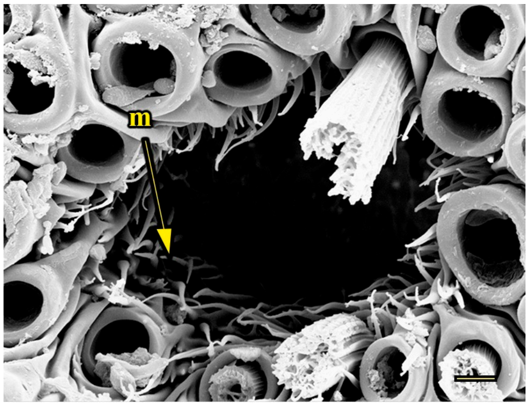

Fig 13. Opening of the vom Rath's organ. *Fountainea ryphea* (Cramer, 1775) (Charaxinae: Anaeini) (♀), m = microtrichia, scale bar 6 μm.

https://doi.org/10.1371/journal.pone.0231486.g013

functional regions: the inner sensory unit, with coeloconic sensilla; and the outer region next to the opening, covered with microtrichia. For some forms these regions are easy to find. In bottle-shaped organs, the sensory unit is in the atrium, while microtrichia cover the neck, as in *Pieris* sp. [4] and *F. ryphea* (Fig 14). The funnel is a frontier between the two morpho-functional regions in tapered organs [27, 28].

The axon associated with vom Rath's organ is adhered to the cuticle in some species, as in *F. ryphea* (Figs 29 and 30) and *M. helenor achillaena* (Figs 31 and 32); whereas in others it is quite evident and free, as in *A. olena* (Fig 34). When the axon is attached to the cuticle it is difficult to characterize. In Lepidoptera, the articulation of the labial palps with the head significantly restricts the passage of hemolymph [41]. Therefore, palps are hollow structures with little tissue that is associated with the cuticle. In species with long labial palps, it would be convenient to have the axon attached to the cuticle. In *A. olena*, the distal palpomerus and the palp itself are small, so the axon associated with vom Rath's organ is typically free.

The studies and reports on non-dytrisian [11–20] and tineid moths [21] do not describe the internal structure and histology of the organ, and they are not considered here for our proposed terminology for the forms of the cavity, the patterns and arrangement of setae, and the type of axon. Descriptions of the internal structure could be difficult because of the small size

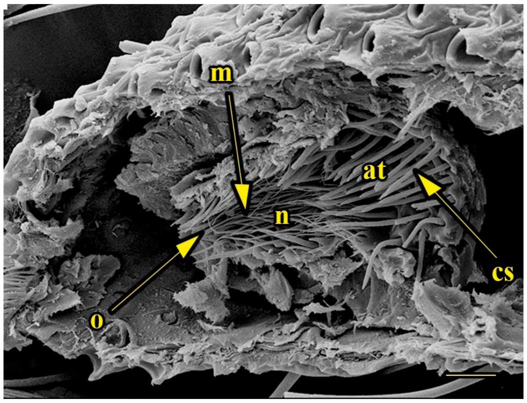

Fig 14. Transverse section of vom Rath's organ. *Fountainea ryphea* (Cramer, 1775) (Charaxinae: Anaeini) (♀), at = atrium, m = microtrichia, n = neck, o = opening, cs = coeloconic sensilla, scale bar 20 μm.

https://doi.org/10.1371/journal.pone.0231486.g014

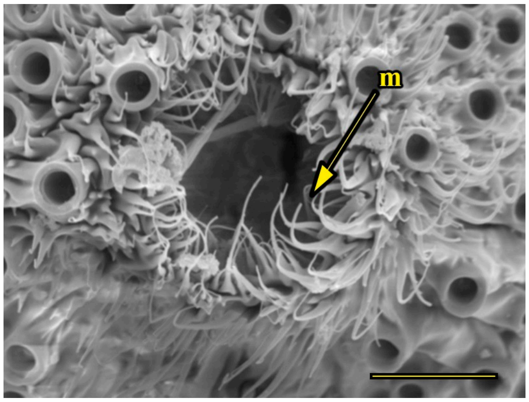

Fig 15. Opening of the vom Rath's organ. *Morpho helenor achillaena* (Hübner, [1819]) (Satyrinae: Morphini) (♂), m = microtrichia, scale bar 20 μm.

https://doi.org/10.1371/journal.pone.0231486.g015

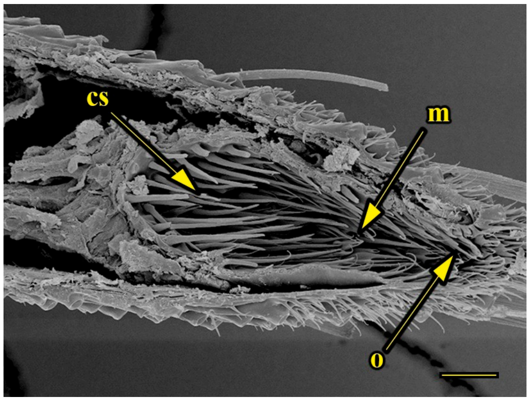

Fig 16. Transverse section of vom Rath's organ. *Morpho helenor achillaena* (Hübner, [1819]) (Satyrinae: Morphini) (♂), m = microtrichia, o = opening, cs = coeloconic sensilla, scale bar 20 μm.

https://doi.org/10.1371/journal.pone.0231486.g016

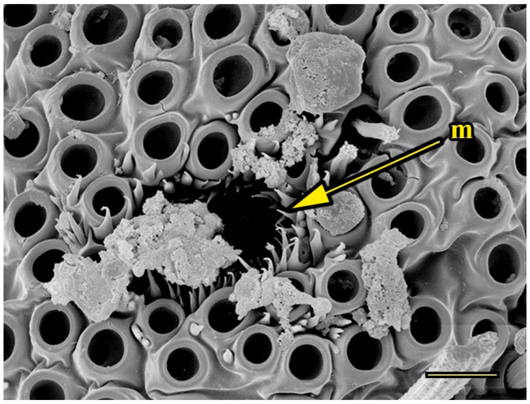

Fig 17. Opening of the vom Rath's organ. *Hamadryas epinome* (Felder & Felder, 1867) (Biblidinae: Ageroniini) (♀), m = microtrichia, scale bar 12 μm.

https://doi.org/10.1371/journal.pone.0231486.g017

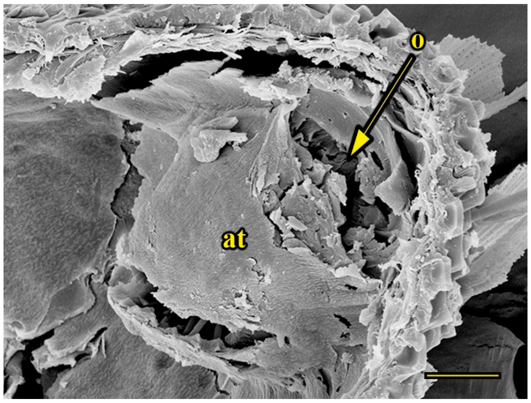

Fig 18. Nearly transverse section of vom Rath's organ. *Hamadryas epinome* (Felder & Felder, 1867) (Biblidinae: Argeroniini) (♀), at = atrium, o = opening, scale bar 20 μm.

https://doi.org/10.1371/journal.pone.0231486.g018

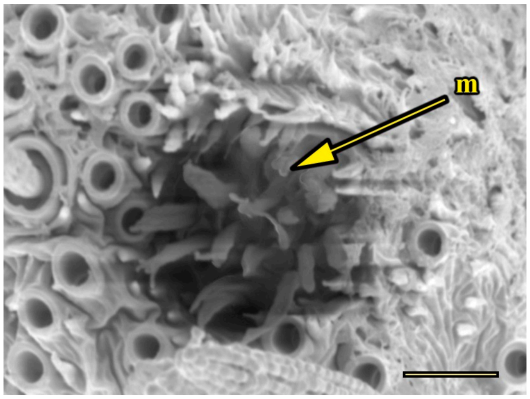

Fig 19. Opening of the vom Rath's organ. *Aeria olena* Weymer, 1875 (Danainae: Ithomiini) (♂), m = microtrichia, scale bar 10 μm.

https://doi.org/10.1371/journal.pone.0231486.g019

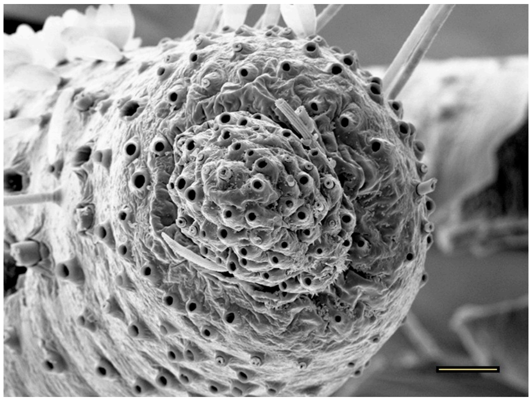

Fig 20. Tip of the distal palpomerus. *Actinote thalia* (Linnaeus, 1758) (Heliconiinae: Acraeini) (♀), scale bar 40 μm.

https://doi.org/10.1371/journal.pone.0231486.g020

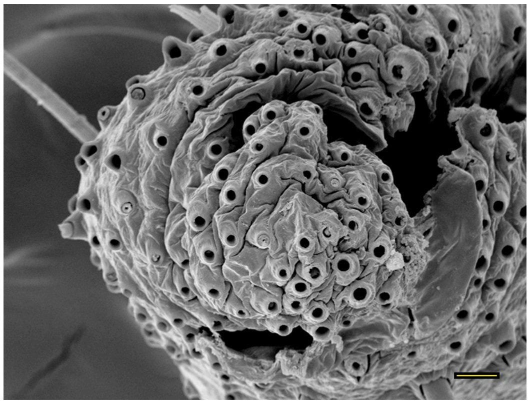

Fig 21. Tip of the distal palpomerus. *Actinote parapheles* Jordan, 1913 (Heliconiinae: Acraeini) (♂), scale bar 20 μm.

https://doi.org/10.1371/journal.pone.0231486.g021

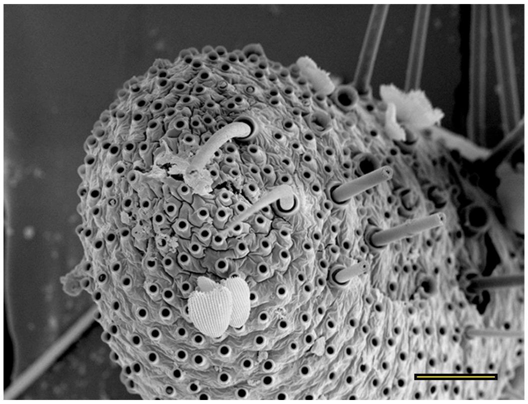

Fig 22. Tip of the distal palpomerus. *Heliconius erato phyllis* (Fabricius, 1775) (Heliconiinae: Heliconiini) (♂), scale bar 40 μm.

https://doi.org/10.1371/journal.pone.0231486.g022

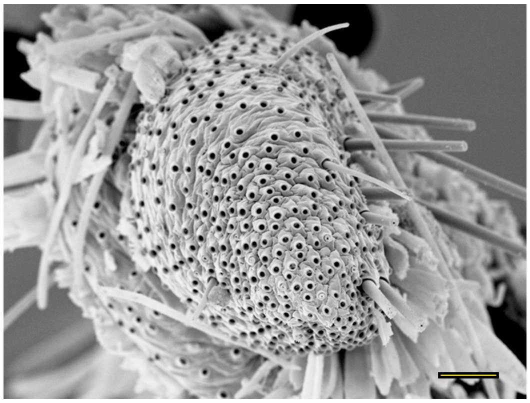

Fig 23. Tip of the distal palpomerus. *Heliconius sara* (Fabricius, 1793) Heliconiinae: Heliconiini) (♂), scale bar 40 μm.

https://doi.org/10.1371/journal.pone.0231486.g023

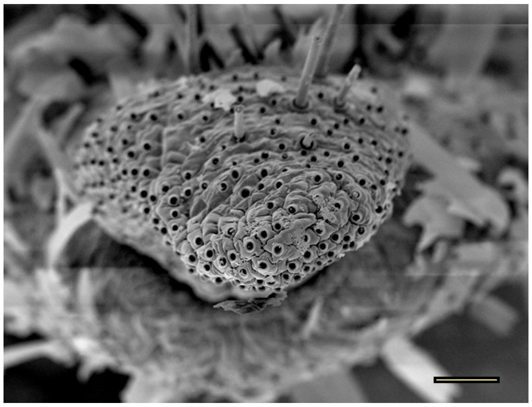

Fig 24. Tip of the distal palpomerus. *Philaethria wernickei* (Röber, 1906) (Heliconiinae: Heliconiini) (♀), scale bar 40 μm.

https://doi.org/10.1371/journal.pone.0231486.g024

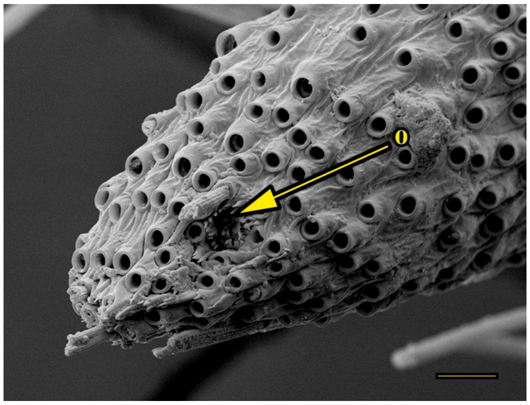

Fig 25. Tip of the distal palpomerus. *Argynnis paphia* (Linnaeus, 1758) (Heliconiinae: Argynnini) (♂), o = opening, scale bar 20 μm.

https://doi.org/10.1371/journal.pone.0231486.g025

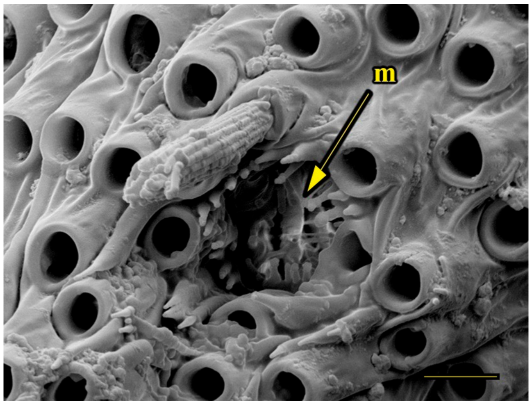

Fig 26. Opening of the vom Rath's organ. *Argynnis paphia* (Linnaeus, 1758) (Heliconiinae: Argynnini) (♂), m = microtrichia, scale bar 8 μm.

https://doi.org/10.1371/journal.pone.0231486.g026

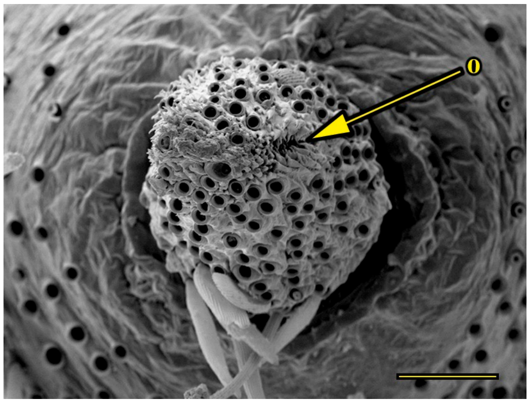

Fig 27. Tip of the distal palpomerus. *Euptoieta hegesia* (Cramer, 1779) (Heliconiinae: Argynnini) (♂), o = opening, scale bar 40 μm.

https://doi.org/10.1371/journal.pone.0231486.g027

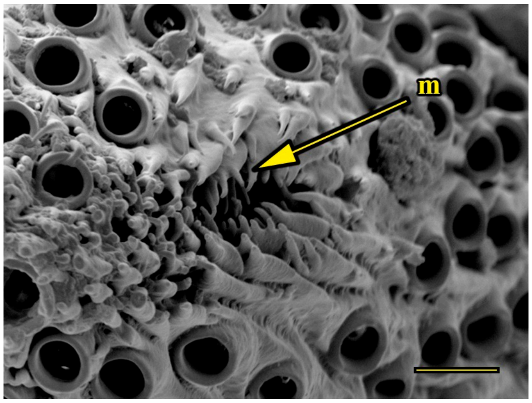

Fig 28. Opening of the vom Rath's organ. *Euptoieta hegesia* (Cramer, 1779) (Heliconiinae: Argynnini) (♀), m = microtrichia, scale bar 9 μm.

https://doi.org/10.1371/journal.pone.0231486.g028

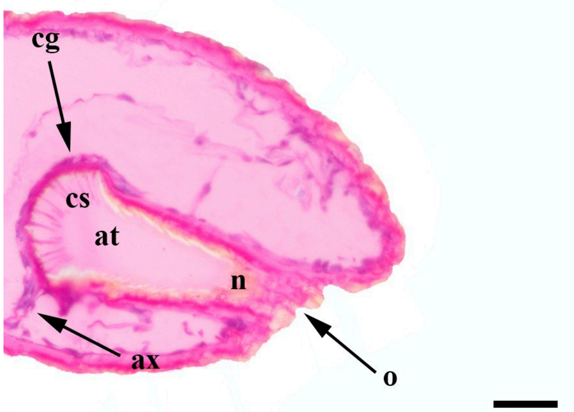

Fig 29. Initial sagittal histological section along the entire length of the vom Rath's organ. *Fountainea ryphea* (Cramer, 1775) (Charaxinae: Anaeini) (♀), at = atrium, ax = axon, cs = coeloconic sensilla, cg = cell groups, n = neck, o = opening, scale bar 50 μm.

https://doi.org/10.1371/journal.pone.0231486.g029

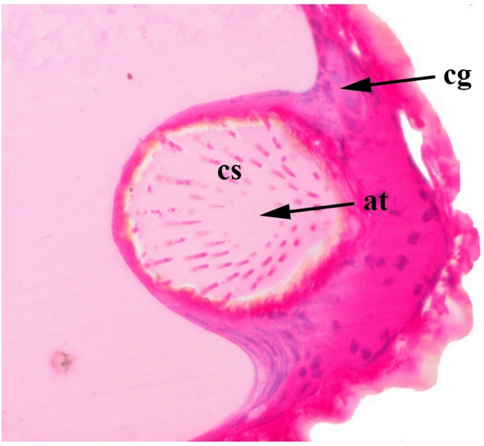

Fig 30. Deeper sagittal histological section along the entire length of the vom Rath's organ. *Fountainea ryphea* (Cramer, 1775) (Charaxinae: Anaeini) (♀), at = atrium, cs = coeloconic sensilla, cg = cell groups, scale bar 20 μm.

https://doi.org/10.1371/journal.pone.0231486.g030

of the species of these groups. For example, we could not successfully section the organs of *H. epinome* and *A. olena*. However, histological descriptions for these groups are relatively easy if fresh specimens are available. It is expected that improved descriptions of vom Rath's organ for these groups will reveal more patterns, especially for non-dytrisian families.

The absence of vom Rath's organ in *Actinote* and Heliconiini

These are the first reports of the absence of vom Rath's organ in members of Lepidoptera. Hicks [2] reported that vom Rath's organ was absent in *Acherontia atropos* (Sphingidae). However, vom Rath [4] described the organ of this species, which is probably sacculiform. In turn, Paluch et al. [42] mentioned that they did not observe vom Rath's organ in *Actinote melanisans* Oberthür, 1917 (Heliconiinae: Acraeini), which agrees with our observations and interpretations for *A. thalia*. However, they did not report the absence but only stated "we did not identify it" (p. 460).

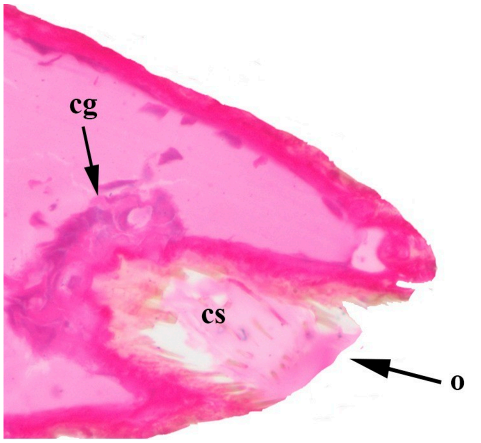

Fig 31. Initial sagittal histological section along the entire length of the vom Rath's organ. *Morpho helenor achillaena* (Hübner, [1819]) (Satyrinae: Morphini) (♂), cs = coeloconic sensilla, cg = cell groups, o = opening, scale bar 20 μm.

https://doi.org/10.1371/journal.pone.0231486.g031

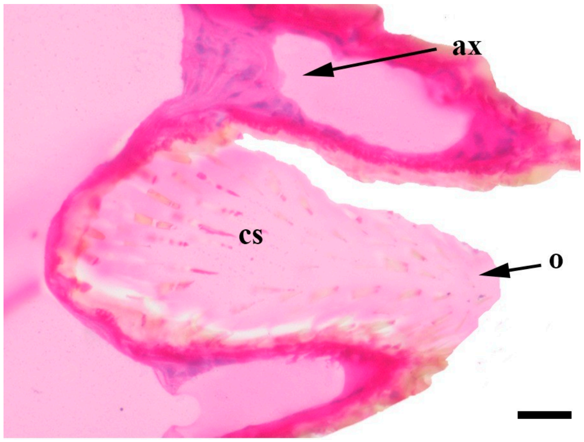

Fig 32. Deeper sagittal histological section along the entire length of the vom Rath's organ. *Morpho helenor achillaena* (Hübner, [1819]) (Satyrinae: Morphini) (♂), ax = axon, cs = coeloconic sensilla, o = opening, scale bar 20 μm.

https://doi.org/10.1371/journal.pone.0231486.g032

Hicks [2] documented the presence of a tubular vom Rath's organ in *Argynnis paphia* (Heliconiinae: Argynnini). This is also confirmed in the present study (Figs 11, 25 and 26), with *Euptoieta hegesia* (Figs 12, 27 and 28) representing the second record for the tribe. However, the organ is absent in the species of *Actinote* examined here, *A. thalia* and *A. parapheles* (Figs 5, 6, 20, 21 and 35), and possibly in *A. melanisans* [42]. The absence of the organ was also documented for different genera of Heliconiini: *A. vanilla*, *H. erato phyllis*, *H. sara*, and *P. wernickei* (Figs 7–10, 22–24 and 36).

Different phylogenetic hypotheses for Heliconiinae have been advanced. Penz & Peggie [43] considered Acraeini as the most basal lineage and sister group of (Heliconiini + (Vagrantini + Argynnini)). In the topology of Freitas & Brown [44], Acraeini is also the basal lineage, but is the sister group of (Argynnini + (*Vindula*/*Cethosia* + Heliconiini)). According to these topologies, the presence of vom Rath's organ in Argynnini would be a reversal event. In contrast, in the hypothesis presented by Wahlberg et al. [45] and corroborated recently by Espeland et al. [46], Argynnini is the basal lineage and sister group of (Vagrantini + (Heliconiini

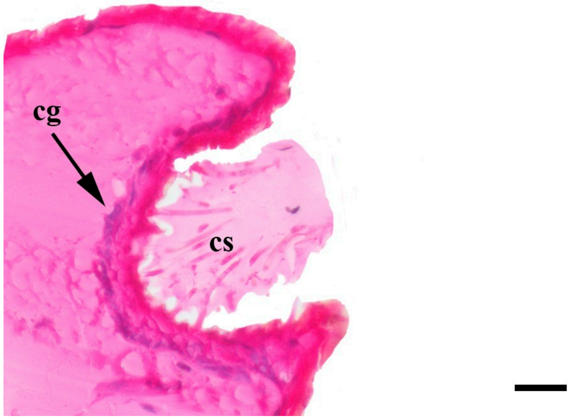

Fig 33. Sagittal histological section along the entire length of the vom Rath's organ. *Hamadryas epinome* (Felder & Felder, 1867) (Biblidinae: Ageroniini) (♀), cs = coeloconic sensilla, cg = cell groups, scale bar 20 μm.

https://doi.org/10.1371/journal.pone.0231486.g033

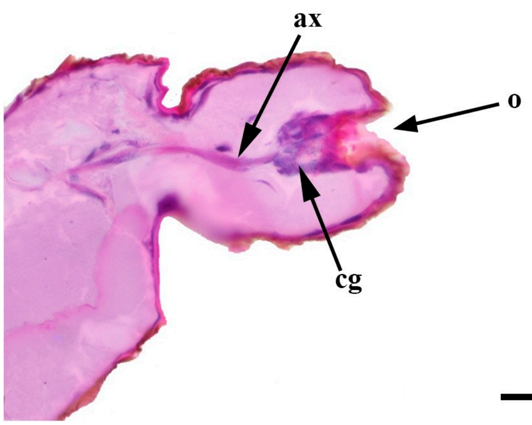

Fig 34. Sagittal histological section along the entire length of the vom Rath's organ. *Aeria olena* Weymer, 1875 (Danainae: Ithomiini) (♂), m = microtrichia, ax = axon, cg = cell groups, o = opening, scale bar 20 μm.

https://doi.org/10.1371/journal.pone.0231486.g034

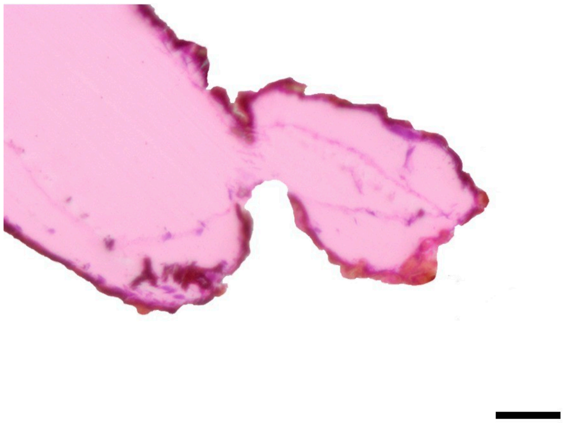

Fig 35. Sagittal histological section along the entire length of the distal palpomerus. *Actinote thalia* (Linnaeus, 1758) (Heliconiinae: Acraeini) (♂), scale bar 50 μm.

https://doi.org/10.1371/journal.pone.0231486.g035

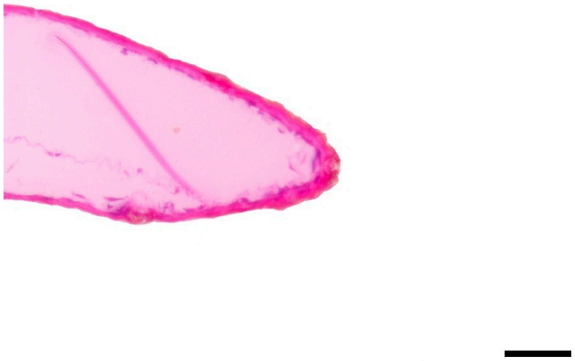

Fig 36. Sagittal histological section along the entire length of the distal palpomerus. *Heliconius erato phyllis* (Fabricius, 1775) (Heliconiinae: Heliconiini) (♂), scale bar 50 μm.

https://doi.org/10.1371/journal.pone.0231486.g036

+ Acraeini + *Vindula*), or of (Heliconiini + (Vagrantini + Acraeini)). Therefore, the absence of vom Rath's organ would be an apomorphy for the clade (Heliconiini + (Acraeini + *Vindula*)) or (Heliconiini + (Vagrantini + Acraeini)).

Inferences about functionality based on the relative size of vom Rath's organ

For species comparisons, two indexes of the relative size of vom Rath's organ were obtained, aiming to interpret the relationship between its development and functionality. The ratio of cavity depth to distal palpomerus length seems to be more informative than the ratio of cavity depth to total palp length. *Fountainea ryphea* and *A*. *olena* have well-developed organs, unlike *M*. *helenor* and especially *H*. *epinome* (Table 1). We are unable to suggest what information theses indexes might furnish about the functionality. Of course, it is not completely certain if these indexes are at least a rough clue to the functionality of vom Rath's organ, but we can consider its presence as a primary indication of the organ's importance.

Vom Rath's organ is involved in CO_2 detection, at least in the Ditrysia clade. Examples of different references are, for Erebidae and Noctuidae [24]; Nymphalidae, Noctuidae, Pieridae, Pyralidae, Saturniidae, and Sphingidae [39]; Noctuidae [40], and Pyralidae [26]. This subject has not been studied in non-ditrysians.

The function of vom Rath's organ for CO_2 detection is well demonstrated as a final stimulus for selecting oviposition sites by *Cactoblastis cactorum* females [26], and there is indirect evidence of this function for *Manduca sexta* [47]. Despite the pronounced sexual dimorphism of the labial palps of *C*. *cactorum* (twice as large in females, and directed forward *vs*. upward in males), we do not know if vom Rath's organ shows sexual dimorphism in this species. In some species with sexual dimorphism in the labial palps, there are no significant differences in vom Rath's organ: *Helicoverpa armigera* (Noctuidae) [27], *Mythimna separata* (Noctuidae) [28], and *Carposina sasakii* (Carposinidae) [29]. Sexual dimorphism was not found in the labial palps or in vom Rath's organ in the four species described here. If the females of these species detect (via vom Rath's organ) and use CO_2 as a stimulus for oviposition, it is not an obvious selective pressure for females, in view of the absence of sexual dimorphism.

Additionally, *M*. *sexta* uses CO_2 gradients to evaluate the amount of nectar in flowers of *Datura wrightii* Regel (Solanaceae) [37, 38]. Based on this precedent, a well-developed vom Rath's organ would be expected in nectarivorous nymphalids because flowers have high respiration rates with high CO_2 gradients. However, the species of Acraeini and Heliconiini that were studied here lack a vom Rath's organ.

Detection of CO_2 gradients has not been studied in frugivorous nymphalid butterflies. However, Sourakov et al. [48], studying *Morpho helenor* (Cramer, 1776) (Satyrinae: Morphini) and *Caligo telamonius* (C. Felder & R. Felder, 1862) (Satyrinae: Brassolini), observed responses of the labial palpi to the volatile substances produced by fermented banana. The authors also noted that these responses are specific to certain volatile compounds other than those detected by the antennae, proboscis and legs, and are always less intense [48]. A wider chemoreceptive function for vom Rath's organ has been proposed [22, 23], but the studies of Bogner et al. [24] and Bogner [39] proved that it is specialized for CO_2 detection in several families. In fact, responses of vom Rath's organ to different chemicals are common, but have certain particularities: i) they are not more intense than the responses to CO_2, ii) they do not depend on the concentration, and iii) they are reduced when CO_2 is removed from the air [39]. Thus, the report of Sourakov et al. [48] needs a reevaluation, following the protocols of Bogner et al. [24] and Bogner [39].

Acknowledgments

In memory of Alfonso Braga Bartolini Salimbeni Vivai for his help with histology at the beginning of our study. The authors thank Leandro Oliveira, Mauricio Martins, Bruno Zilberman, Lara Guimarães, Márcio Valentim Cruz, the students of the Laboratório de Borboletas da Universidade Estadual de Campinas, and the students of the Laboratório de Histofisiologia Evolutiva da Universidade de São Paulo for technical support. Renato de Oliveira e Silva, Rafael Dell'Erba and Ernesto Aranda also made valuable contributions. Janet Reid revised and edited the manuscript. JL thanks Sueli Felizinai for helping in numerous ways and making possible this project.

Author Contributions

Conceptualization: Joel Lastra-Valdés, José Roberto Machado Cunha da Silva, Marcelo Duarte.

Data curation: Joel Lastra-Valdés.

Formal analysis: Joel Lastra-Valdés, José Roberto Machado Cunha da Silva, Marcelo Duarte.

Funding acquisition: Marcelo Duarte.

Investigation: Joel Lastra-Valdés, José Roberto Machado Cunha da Silva.

Methodology: Joel Lastra-Valdés, José Roberto Machado Cunha da Silva.

Writing – original draft: Joel Lastra-Valdés.

Writing – review & editing: Joel Lastra-Valdés, José Roberto Machado Cunha da Silva, Marcelo Duarte.

References

1. Kristensen NP, Scoble MJ, Karsholt O. Lepidoptera phylogeny and systematics: the state of inventorying moth and butterfly diversity. Zootaxa 2007; 1668: 699–747.
2. Hicks JB. On certain sensory organs in insects, hitherto undescribed. Trans Linn Soc Lond. 1860; 23:139–155.
3. vom Rath O. Über die Hautsinnesorgane der Insecten. Zool Anz. 1887; 10: 627–631, 645–649.
4. vom Rath O. Über die Hautsinnesorgane der Insecten. Z Wiss Zool. 1888; 46: 413–454.
5. Orfila RN. Las especies argentinas de "Prepona" Boisduval (Lep: Nymph.). Rev Mus Argent Cienc Nat Bernardino Rivadavia Inst Nac Invest Cienc Nat. 1950; 1: 273–321.
6. Duarte M, Casagrande MM, Mielke OHH. Morfologia externa do adulto de Hemiargus hanno (Stoll) (Lepidoptera, Lycaenidae, Polyommatinae, Polyommatini). I. Cabeça. Rev Bras Zool. 2001; 18: 225–238.
7. Bizarro JMS, Casagrande MM, Mielke OHH. Morfologia externa de Thyridia psidii cetoides (Rosenberg & Talbot). I. Cabeça e apêndices (Lepidoptera, Nymphalidae, Ithomiinae). Rev Bras Zool. 2003; 20: 279–284.
8. Mielke CGC, Mielke OHH, Casagrande MM. Estudo comparado da morfologia externa de Zaretys itys itylus (Westwood) e Agrias claudina annetta (Gray) (Lepidoptera, Nymphalidae, Charaxinae) I. Cabeça, apêndices e região cervical. Rev Bras Zool. 2004; 21: 357–369.
9. Camargo AJA, Casagrande MM, Mielke OHH, Furtado E. Morfologia externa do adulto de Almeidaia aidade Mielke & Casagrande (Lepidoptera, Saturniidae, Arsenurinae, Almeidaiini). I. Cabeça, apêndices e região cervical. Rev Bras Zool. 2005; 22: 619–622.
10. Leite LAR, Casagrande MM, Mielke OHH. External morphology of the adult of Heraclides anchisiades capys (Hübner, [1809]) (Lepidoptera–Papilionidae) I. Head, cephalic appendages and cervical region. Braz Arch Biol Technol. 2010; 53: 1119–1126.
11. Kristensen NP. The skeletal anatomy of the heads of adult of Mnesarchaeidae and Neopseustidae (Lep., Dacnonypha). Entomol Medd. 1968; 36: 137–151.

12. Davis DR. Systematics and zoogeography of the family Neopseustidae with the proposal of a new superfamily (Lepidoptera: Neopseustoidea). Smithson Contrib Zool. 1975; 210: 1–45.

13. Faucheux MJ. Les sensilles basiconiques gaufrées à base doublé ou triple de l'antenne du papillon sud-américain, Apoplania valdiviana Davis & Nielsen, 1984 (Lepidoptera: Neupseustidae): distribuition et importance numérique. Bull Soc Sci Nat Ouest Fr. 2005; 27: 1–18.

14. Faucheux MJ. Mouthparts and associated sensilla of a South American moth, Synempora andesae (Lepidoptera: Neupseustidae). Rev Soc Argent. 2008; 67: 21–33.

15. Chauvin G, Faucheux M. Les pieces buccales et leurs recepteurs sensoriels chez l'imago de Micropterix calthella L. (Lepidoptera: Micropterigidae). Int J Insect Morphol Embryol. 1981; 10: 425–439.

16. Kristensen NP. Skeleton and muscle. In: Kristensen NP, editor. Lepidoptera, moths and butterflies. Volume 2: Morphology, physiology, and development. In: Fischer M, editor. Handbuch der Zoologie/ Handbook of Zoology. Band/Volume IV Arthropoda: Insecta: Moths and Butterflies. Berlin: Walter de Gruyter; 2003. pp. 39–123.

17. Nielsen ES, Davis DR. A revision of the Neotropical Incurvariidae s. str., with the description of two new genera and two new species (Lepidoptera: Incurvarioidea). Steenstrupia 1981; 7: 25–57.

18. Davis DR. A new family of monotrysian moths from austral South America (Lepidoptera: Palaephatidae) with a phylogenetic review of the Monotrysia. Smithson Contrib Zool. 1986; 434: 1–202.

19. Faucheux MJ. Heterobathmia pseuderiocrania (Heterobathmiina): mouthparts, mouthpart sensilla, and comparison with other non-glossatan suborders (Insecta, Lepidoptera). Rev Mus Argentino Cienc Nat. 2005; 7: 57–65.

20. Davis DR, Gentili P. Andesianidae, a new subfamily of monotrysian moths (Lepidoptera: Andesianoidea) from austral South America. Invertebr Syst. 2003; 17: 15–26.

21. Faucheux MJ, Chauvin G. Les pièces buccales des adultes de cinq Lépidoptères Tinéides keratophages communs dans l'ouest de la France. II—Les récepteurs sensoriels des palpes labiaux. Bull Soc Sci Nat Ouest Fr. 1980; 2: 4–15.

22. Lee JK, Selzer R, Altner H. Lamellated outer dendritic segments of a chemoreceptor within wall-pore sensilla in the labial palp-pit organ of the butterfly, Pieris rapae L. (Insecta, Lepidoptera). Cell Tissue Res. 1985; 240: 333–342.

23. Kent KS, Harrow ID, Quartararo P, Hildebrand JG. An accessory olfactory pathway in Lepidoptera: the labial pit organ and its central projections in *Manduca sexta* and certain other sphinx moths and silk moths. Cell Tissue Res. 1986; 245: 237–245. https://doi.org/10.1007/bf00213927 PMID: 3742559

24. Bogner F, Boppre M, Ernst K-D, Boeckh J. CO2 sensitive receptors on labial palps of Rhodogastria moths (Lepidoptera, Arctiidae): physiology, fine-structure and central projection. J Comp Physiol A 1986; 158: 741–749. https://doi.org/10.1007/bf01324818 PMID: 3090241

25. Faucheux MJ. Morphology and distribution of sensilla on the cephalic appendages, tarsi and ovipositor of the European sunflower moth, Homoeosoma nebulella Den. & Schiff. (Lepidoptera: Pyralidae). Int J Insect Morphol Embryol. 1991; 20: 291–307.

26. Stange G, Monro J, Stowe S, Osmond CB. The CO_2 sense of the moth *Cactoblastis cactorum* and its probable role in the biological control of the CAM plant *Opuntia stricta*. Oecologia 1995; 102: 341–352. https://doi.org/10.1007/BF00329801 PMID: 28306845

27. Zhao XC, Tang QB, Berg BG, Liu Y, Wang YR, Yang FM, et al. Fine structure and primary sensory projections of sensilla located in the labial-palp pit organ of Helicoverpa armigera (Insecta). Cell Tissue Res. 2013; 353: 399–408. https://doi.org/10.1007/s00441-013-1657-z PMID: 23736380

28. Dong JF, Liu H, Tang QB, Liu Y, Zhao XC, Wang GR. Morphology, type and distribution of the labial-palp pit organ and its sensilla in the oriental armyworm, Mythimna separata (Lepidoptera: Noctuidae). Acta Entomol Sin. 2014; 57: 681–687.

29. Chen J, Hua B. Sexual dimorphism of adult labial palps of the peach fruit moth Carposina sasakii Matsumura (Lepidoptera: Carposinidae) with notes on their sensilla. Acta Zool. 2016; 97: 42–48.

30. Song YQ, Sun HZ, Wu JX. Ultrastructural characteristics of the proboscis and the labial palp pit organ in the oriental fruit moth, Grapholita molesta. Bull Insectology 2016; 69: 59–66.

31. Hennig W. Phylogenetic systematics. Annu Rev Entomol. 1965; 10: 97–166.

32. Kristensen NP. Studies on the morphology and systematics of primitive Lepidoptera (Insecta). Steenstrupia 1984; 10: 141–191.

33. Brown Jr KS. Borboletas da Serra do Japi: diversidade, habitats, recursos alimentares e variação temporal. In: Morellato LPC. (Org.) História natural da Serra do Japi: ecologia e preservação de uma área florestal no Sudeste do Brasil. Campinas, Editora Unicamp. 1992; 142–186.

34. Rohlf FJ. tpsDig–thin plate splines digitizer, version 2.11. New York: State University of New York at Stony Brook; 2001.

35. McDowell EM, Trump BF. Histologic fixatives suitable for diagnostic light and electron microscope. Arch Pathol Lab Med. 1976; 100: 405–414. PMID: 60092

36. Nation JL. A new method using hexamethyldisilazane for preparation of soft insect tissue for scanning electron microscopy. Stain Technol. 1983; 58: 347–351. https://doi.org/10.3109/10520298309066811 PMID: 6679126

37. Guerenstein PG, Yepez EA, van Haren J, Williams DG, Hildebrand JG. Floral CO_2 emission may indicate food abundance to nectar-feeding moths. Naturwissenschaften 2004; 91: 329–333. https://doi.org/10.1007/s00114-004-0532-x PMID: 15257387

38. Thom C, Guerenstein PG, Mechaber WL, Hildebrand JG. Floral CO_2 cues reveal flower profitability to moths. J Chem Ecol. 2004; 30: 1285–1288. https://doi.org/10.1023/b:joec.0000030298.77377.7d PMID: 15303329

39. Bogner F. Sensory physiological investigation of carbon-dioxide receptors in Lepidoptera. J Insect Physiol. 1990; 36: 951–957.

40. Stange G. High-resolution measurement of atmospheric carbon dioxide concentration changes by the labial palp organ of the moth Heliothis armigera (Lepidoptera, Noctuidae). J Comp Physiol A 1992; 171: 317–324.

41. Schmitt JB. The feeding mechanism of adult Lepidoptera. Smithsonian Misc Collect. 1938; 97: 1–27.

42. Paluch M, Casagrande MM, Mielke OHH. Morfologia externa do adulto de Actinote melanisans (Nymphalidae: Heliconiinae: Acraeini). Rev Bras Zool. 2008; 25: 456–478.

43. Penz CM, Peggie D. Phylogenetic relationships among Heliconiinae genera based on morphology (Lepidoptera: Nymphalidae). Syst Entomol. 2003; 28: 451–479.

44. Freitas AVL, Brown KS Jr. Phylogeny of the Nymphalidae (Lepidoptera). Syst Biol. 2004; 53: 363–383. https://doi.org/10.1080/10635150490445670 PMID: 15503668

45. Wahlberg N, Weingartner E, Nylin S. Towards a better understanding of the higher systematics of Nymphalidae (Lepidoptera: Papilionoidea). Mol Phylogenet Evol. 2003; 28: 473–484. https://doi.org/10.1016/s1055-7903(03)00052-6 PMID: 12927132

46. Espeland M, Breinholt J, Willmott KR, Warren AD, Vila R, Toussaint EFA et al. A comprehensive and dated phylogenomic analysis of butterflies. Curr Biol 2018; 28: 770–778. https://doi.org/10.1016/j.cub.2018.01.061 PMID: 29456146

47. Abrell L, Guerenstein PG, Mechaber WL, Stange G, Christensen TA, Nakanishi K, Hildebrand JG. Effect of elevated atmospheric CO2 on oviposition behavior in Manduca sexta moths. Global Change Biol. 2005; 11: 1272–1282.

48. Sourakov A, Duehl A, Sourakov A. Foraging behavior of the Blue *Morpho* and other tropical butterflies: the chemical and electrophysiological basis of olfactory preferences and the role of color. Psyche 2012; 2012: 1–10.
